# Rapid emergence of resistance to antiretroviral treatment after undisclosed prior exposure: A case report

**DOI:** 10.4102/sajhivmed.v20i1.965

**Published:** 2019-07-30

**Authors:** Theresa M. Rossouw, Gisela van Dyk, Gert van Zyl

**Affiliations:** 1 Department of Immunology, University of Pretoria, Pretoria, South Africa; 2 Division of Medical Virology, Stellenbosch University and National Health Laboratory Service, Bellville, South Africa

**Keywords:** HIV drug resistance, Antiretroviral therapy, Undisclosed prior treatment

## Abstract

**Introduction:**

Patients who disengaged from care may present as therapy naïve for antiretroviral treatment (ART) initiation at a different site, without being recognised as being at an increased risk of rapid treatment failure and HIV drug resistance.

**Patient presentation:**

A 43-year-old woman, who gave no prior history of ART, was initiated on a standard first-line regimen of TDF, FTC and EFV. She had a poor response to treatment with evidence of treatment failure at 12 months.

**Management and outcome:**

HIV-1 drug resistance tests showed no pre-treatment HIVDR mutations, but revealed high-level drug resistance to all component drugs at 12 months. On investigation, viral load (VL) was recorded in 2012 and 2013, providing evidence of prior ART use.

**Conclusion:**

Linkage of patient therapy and laboratory information to unique patient identifiers may allow health-care workers to identify patients who previously received ART and disengaged from care. This will enable differentiated care when these patients reinitiate ART, which should involve expedited VL testing and more rapid transition to definitive second-line ART.

## Introduction

HIV drug resistance (HIVDR) is a major public health concern, especially in the context of a large treatment programme. Patients who disengage from care and then return to the health-care system without disclosing previous antiretroviral therapy (ART) are at increased risk of having pre-existing drug resistance. Unfortunately, patients rarely report prior ART use, and health-care workers do not routinely ask and record this. All drugs in the current first-line regimen (TDF, FTC and EFV) have low genetic barriers, and hence, one or two mutations lead to diminished activity, which can affect entire drug classes. HIV drug resistance testing is not currently available in the South African public sector for patients initiating or failing first-line ART. Here we describe a case of a patient with undisclosed prior exposure to ART who had a complex HIVDR pattern at treatment failure and highlight potential risk factors for rapid HIVDR emergence.

## Case description

**FIGURE 1:**
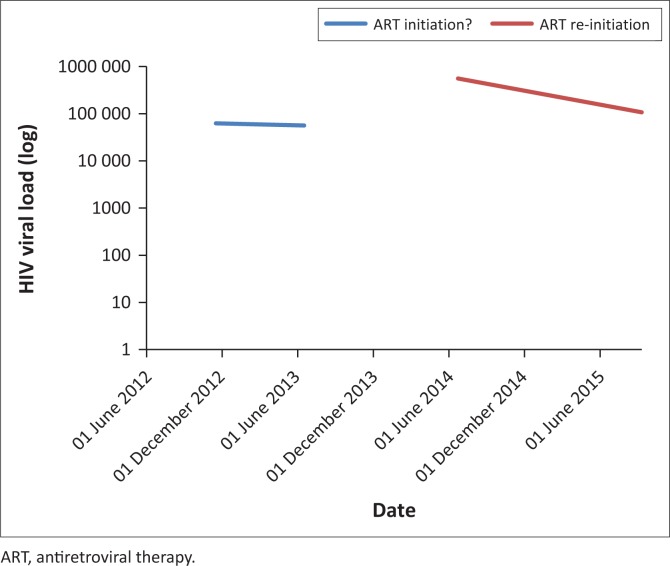
HIV viral load results over time.

A 43-year-old woman presented to a clinic in Tshwane, South Africa, on 23 July 2014. She tested HIV-positive and had a CD4 count of 14 cells/µL and HIV-1 viral load (VL) of 560 000 copies/mL. She gave no prior history of ART and was initiated on a first-line regimen of TDF, FTC and EFV on 06 August 2014. She attended all her visits on time and reported good adherence, but had a poor response after 12 months of treatment: CD4 53 cells/µL and VL 186 000 copies/mL. The 6-month VL had not been performed. As part of a research project (ethics approval 469/2013), she had a drug resistance test (DRT) with a validated in-house Sanger-based sequencing method^1^ before the initiation of ART, which showed no HIVDR mutations, and again after 12 months, which revealed six nucleoside/nucleotide reverse transcriptase inhibitor (NRTI) and three non-nucleoside reverse transcriptase inhibitor (NNRTI) mutations (Tables 1 and 2). A search of the NHLS database for evidence of prior HIV-related testing revealed two VL results (2012 and 2013), which precede her ART-initiation date (Figure 1).

**TABLE 1:** Drug resistance report at 12 months.

**Drug resistance interpretation: RT**	
NRTI Resistance Mutations	A62V, K65R, V75I, Y115F, M184V, K219E
NNRTI Resistance Mutations	L100I, K103N
Other Mutations	V90I
**Nucleoside reverse transcriptase inhibitors**	
abacavir (ABC)	High-level Resistance
zidovudine (AZT)	Susceptible
emtricitabine (FTC)	High-level Resistance
lamivudine (3TC)	High-level Resistance
tenofovir (TDF)	High-level Resistance
**Non-nucleoside reverse transcriptase inhibitors**	
doravirine (DOR)	Intermediate Resistance
efavirenz (EFV)	High-level Resistance
etravirine (ETR)	Intermediate Resistance
nevirapine (NVP)	High-level Resistance
rilpivirine (RPV)	High-level Resistance

*Source*: Adapted from the Stanford database^2^

Note: The drug resistance profile reveals a mixed pattern consisting of the classic XTC-related mutation, M184V/I; two TDF-associated mutations, Y115F and K65R; one accessory thymidine-associated mutation (TAM) related to decreased susceptibility to d4T and AZT, namely K219Q/E; and two accessory mutations, A62V and V75I, that have been described with K65R^3^ but mostly occur in combination with the multi-NRTI resistance mutation Q151M. The two NNRTI mutations, L100I and K103N, give broad-spectrum resistance to the NNRTI class, including intermediate resistance to the second-generation NNRTIs, while V90I is a polymorphic accessory mutation selected by NNRTIs and is associated with minimal reduction in susceptibility to this class.^2^

**TABLE 2:** Mutation-penalty score for the reverse transcriptase inhibitors.

Mutation Scoring: RT					
NRTI	ABC	AZT	FTC	3TC	TDF
A62V	5	5	5	5	5
K65R	45	−15	30	30	60
V75I	5	5	5	5	5
Y115F	60	0	0	0	15
M184V	15	−10	60	60	−10
K219E	5	10	0	0	5

**Total**	**135**	**−5**	**100**	**100**	**80**

**NNRTI**	**DOR**	**EFV**	**ETR**	**NVP**	**RPV**

L100I	15	60	30	60	60
L100I + K103N	15	0	0	0	0
K103N	0	60	0	60	0

**Total**	**30**	**120**	**30**	**120**	**60**

*Source*: Adapted from the Stanford database^2^

Note: This combination of mutations leaves only AZT as a viable NRTI.

RT, reverse transcriptase; NRTI, nucleoside/nucleotide reverse transcriptase inhibitors; ABC, abacavir; AZT, zidovudine; FTC, emtricitabine; 3TC, lamivudine; TDF, tenofovir disoproxil fumarate; NNRTI, non-nucleoside reverse transcriptase inhibitor; DOR, doravirine; EFV, efavirenz; ETR, etravirine; NVP, nevirapine; RPV, rilpivirine.

### Ethical consideration

This study was part of a research project that had been approved by the Research Ethics Committee of the Faculty of Health Sciences of the University of Pretoria (ethics approval number 469/2013).

## Discussion

This case study describes the rapid emergence of high-level, dual-class HIVDR in a patient with undisclosed prior ART use. Having nine *reverse transcriptase* mutations within 12 months is much faster than the reported rate of emergence.^4^ This together with the unsuppressed VL during 2012 and 2013 suggests prior HIVDR mutation selection, which waned to below the detection threshold of population sequencing (± 20%) when DRT was performed in 2014. Thereafter, it rapidly re-emerged upon reintroduction of ART.

Prior undisclosed use of ART is a common problem, requiring vigilance, especially in health sectors where patients are mobile and do not have unique identifiers, as is the case in South Africa. Recent data from Botswana showed that 136/951 (14%) HIV-infected participants who reported no prior ART use had a baseline VL of < 400 copies/mL, and of these, 39% had detectable ART levels in plasma.^5^ A study from Khayelitsha reported that 23% of patients disengaged from care for at least 6 months and that ~50% of these returned to care in the medium term.^6^ Almost 1/3 of adults restarting first-line ART with prior ART exposure harbour resistant virus with women twice as likely as men to have resistance because of previous ART exposure during pregnancy.^7^ Patients with multiple treatment interruptions and/or pre-treatment drug resistance (PDR) are at an increased risk of virological failure.^8,9,10,11^ This patient had seven different laboratory numbers, making linking her previous results complicated. Patients with prior ART may benefit from differentiated care with specialised adherence support, an early VL^12^ (at 4 months), DRT in case of a suboptimal VL response and rapid change to a second-line regimen. The absence of a 6-month VL in this patient made prompt action impossible. She had a number of risk factors for treatment failure, namely previous ART, high VL and previous adherence issues, which could have alerted the astute clinician had this information been available.
